# Impact of Contextual Factors on the Attendance and Role in the Evidence-Based Chronic Disease Prevention Programs Among Primary Care Practitioners in Shanghai, China

**DOI:** 10.3389/fpubh.2021.666135

**Published:** 2022-02-02

**Authors:** Xin Liu, Xin Gong, Xiang Gao, Zhaoxin Wang, Sheng Lu, Chen Chen, Hua Jin, Ning Chen, Yan Yang, Meiyu Cai, Jianwei Shi

**Affiliations:** ^1^School of Public Health, Shanghai Jiaotong University School of Medicine, Shanghai, China; ^2^Department of Heart Failure, Shanghai East Hospital Affiliated to Tongji University School of Medicine, Shanghai, China; ^3^Shanghai General Practice and Community Health Development Research Center, Shanghai, China; ^4^Department of Orthopedics (Spine Surgery), The First Affiliated Hospital of Wenzhou Medical University, Wenzhou, China; ^5^Shanghai Jing'an District Jiangning Road Community Health Service Center, Shanghai, China; ^6^Department of General Practice, Yangpu Hospital, Tongji University School of Medicine, Shanghai, China; ^7^Academic Department of General Practice, Tongji University School of Medicine, Shanghai, China; ^8^School of Medicine, Tongji University, Shanghai, China; ^9^Obstetrics and Gynecology Hospital of Fudan University, Shanghai, China

**Keywords:** evidence-based practice (EBP), chronic disease, general practitioners, primary care (MeSH), preventative interventions

## Abstract

**Background:**

The implementation of evidence-based approaches by general practitioners (GPs) is new in the primary care setting, and few quantitative studies have evaluated the impact of contextual factors on the attendance of these approaches.

**Methods:**

In total, 892 GPs from 75 community healthcare centers (CHCs) in Shanghai completed our survey. We used logistic regression to analyze factors affecting the number of evidence-based chronic disease programs attended by GPs and whether they had held the lead position in such a program.

**Results:**

A total of 346 (38.8%) of the practitioners had never participated in any evidence-based chronic disease prevention (EBCDP) program. The EBCDP interventions in which the GPs had participated were predominantly related to hypertension, diabetes, and cardiovascular disease. However, the proportion of GPs in the lead role was relatively low, between 0.8% (programs involving prevention and control of asthma) and 5.0% (diabetes). Organizational factors and areas were significantly associated with evidence-based practices (EBPs) of the GP, while monthly income and department were the most significantly related to GPs who have the lead role in a program. The results indicated that GPs who had taken the lead position had higher scores for policy and economic impeding factors. GPs who were men, had a higher income, and worked in prevention and healthcare departments and urban areas were more likely to take the lead position.

**Conclusion:**

Evidence-based programs for chronic diseases should be extended to different types of diseases. Personal, organizational, political, and economic factors and the factors of female sex, lower income, department type, and suburban area environment should be considered to facilitate the translation of evidence to practice.

## Background

The emergence of chronic diseases is becoming a predominant global health challenge, and preventing and controlling chronic diseases have gradually become a long-term health policy project in many countries and are included in the Healthy China 2030 strategic plan (1). After China's New Health Reform of 2009, the government accelerated the construction of primary healthcare institutions and expanded the team of general practitioners (GPs) facing the pressure of an aging population, replacing the situation of secondary and tertiary hospitals taking the main role of both medical and prevention service provision (2–4). In China, primary healthcare institutions consisted of community healthcare centers (CHCs) in cities, township health centers in countries, and village clinics in villages, which covered 55% of outpatient care (4.4 billion visits) in 2016 (3). Among them, the CHCs in cities have developed the fastest and contain a sound structure, usually with departments, such as Western medicine, traditional Chinese medicine, and preventive healthcare. Usually, in large cities, such as Shanghai, CHCs provide services for the local residents, ranging in number from ~50,000 to 150,000 (3). Usually, CHCs take the responsibility of preventative healthcare instead of larger hospitals, as the New Health Policy requires (4, 5). However, the efficiency of preventative healthcare is still not optimal among CHCs and other primary care institutions in China (3).

Existing studies have indicated that population-based preventative interventions through the application of scientific evidence can significantly increase work efficiency (6, 7). For instance, Kennedy et al. enrolled 314 children in an intervention group and 276 children in a control group in a study that assessed the existing evidence of risk factors for asthma and effective interventions to reduce asthma morbidity in vulnerable populations. After 12 months of the Community Healthcare for Asthma Management and Prevention of Symptoms (CHAMPS) intervention [creating safe sleeping zones, removing cockroaches, and rodents in the home (8)], the symptomatic asthma days of the children in the intervention group were significantly reduced compared with those of the control group (9). The successful translation of asthma evidence to intervention practice has reduced the morbidity of asthma and increased work efficiency.

In recent years, evidence-based practices (EBPs) for chronic disease prevention have been increasingly encouraged among health practitioners (10). In Western countries, studies on impediments to EBPs related to chronic diseases have been conducted in state and local health departments to facilitate EBPs of the public health practitioners (11, 12). Jacobs et al. investigated the extent to which personal and organizational factors impeded evidence-based decision-making and found that experts were considered the largest personal barrier and that incentives, funding, and legislation were considered the greatest organizational barriers (13). Regarding external environmental and policy factors, research by Dodson et al. suggested that a lack of training, time, and funds were the main barriers to the use of evidence-based methods, and political, structural, and management challenges were the secondary barriers (11). Furtado et al. explored the political contextual factors that impact the implementation of evidence-based chronic disease prevention (EBCDP) (14). However, the existing research on the influencing factors has mostly been qualitative, and few quantitative studies have comprehensively examined the impeding factors, namely, at the personal, organizational, external environmental, policy, and economic levels, to thoroughly understand the crucial reasons for resistance. In China, EBPs have not been widely promoted among GPs in local CHCs, who play a primary role in chronic disease prevention and control (2), and little is known regarding the implementation of EBPs and the factors impeding it (14–16).

In this study, we conducted a quantitative investigation to assess the EBPs of GPs that include the number of evidence-based programs they had participated in and the role they played in such programs. Additionally, we examined the possible comprehensive impeding factors for EBPs to emphasize measures to promote EBP implementation.

## Materials and Methods

### Data Source

We used a random number generator to select 39 suburban and 39 urban CHCs from 246 CHCs in Shanghai, but 3 suburban CHCs did not participate in our study. To make the results reasonable, we randomly selected 6 junior GPs, 6 mid-level GPs, and 1 senior GP in each selected CHC according to the composition of GPs in CHCs. From April to July 2019, we distributed a total of 975 questionnaires, of which 892 valid questionnaires were returned.

### Measurement

The questionnaire was adapted from a study comparing the use of EBCDP processes (17, 18). At the beginning of the questionnaire, we explained the purpose of the questionnaire and relevant concepts to the respondents (EBCDP, evidence-based programs, etc.). The questionnaire consisted of four sections: demographics (10 items), practice and application of EBPs for various chronic diseases (39 items, 24 multiple-choice items, and 15 7-point Likert scale items to measure the number of evidence-based programs they had participated in and the role they played in such programs), and various contextual impediments to EBPs (26 items, 25 7-point Likert scale items) from Brownson et al.'s tool at Washington University. Before distributing the questionnaire, we conducted a pilot test to ensure feasibility. The Cronbach's α of the total scale was 0.980, and the Spearman-Brown coefficient was 0.912. Our questionnaire was proven to have good reliability and validity (10).

### Independent Variables

#### Demographics

In our study, the demographics consisted of gender, age, education, employment title, working years, monthly income, department, and area. Educational qualifications were divided into associate's degree or below, bachelor's degree, and master's degree or higher. GPs were awarded junior, mid-level, and senior titles according to their work experience and achievements. The GPs worked in CHC departments of general medicine (Western medicine), prevention and healthcare, general practice (traditional Chinese medicine), and other departments, such as medical technology and rehabilitation. The urban and suburban areas were defined according to the geographic regions where CHCs were located.

#### Contextual Impeding Factors

The possible influencing factors comprised four aspects, each of which contained various questions. As shown in Table 1, the first aspect is personal that includes the lack of skills to find evidence, lack of skills to develop evidence-based interventions, and lack of time. The second aspect was organizational, such as work atmosphere, leadership engagement, and lack of access to evidence. External environmental factors were the third factor, referring mainly to impediments caused by the cooperation of local community residents. The fourth aspect was policy and economic factors, such as financial support and policy support from the government. The questions in all four aspects were answered on 7-point Likert scales. The respondents were asked to indicate how much they agreed with the arguments, with “1” representing “strongly disagree” and “7” representing “strongly agree”. For each aspect, we calculated the mean of the subordinating variables to indicate the barrier, and a higher score represented a more severe situation.

**Table 1:** Description of the questionnaires.

**Section**	**Items**	**Sub-items**
Demographics		Age, gender, education, position, working years, monthly income, department, area
Contextual impediments	Personal factors	Low value of evidence-based approaches; lack of skills to find evidence and develop evidence-based interventions; lack of decision-making authority; not enough time
	Organizational factors	Poor understanding of evidence-based approaches; poor culture/climate; lack of leadership support; lack of internal policy; not enough funding or staff
	External environmental factors	Distrust of scientific data in the populations served; local residents' perception conflicts; insufficient relevant evidence
	Policy and economic factors	Not enough financial support from the government; funding changes as political leadership changes; policy climate; no existing policies to support
Practice and application of EBPs on various chronic diseases	Number of evidence-based programs in which GPs participated	How many evidence-based chronic disease prevention programs have you attended?
	The role GPs played in such programs	What is your role when attending these programs? Leader, main performer, participant, assessor, none

#### Dependent Variables

In this study, we measured attendance and role of GPs in evidence-based programs through two variables: (1) the number of chronic disease programs participated in by GPs and (2) whether they held the leadership role in such a program. Evidence-based chronic disease programs, usually funded and initiated by provincial or regional CDCs or health administrative institutions, use evidence-based methods to prevent the onset of chronic diseases and manage the population with chronic diseases. Different from ordinary chronic disease programs, evidence-based chronic disease programs require GPs to use evidence to intervene in community residents. In a specific program, the person with the lead role is usually the founder of the program, and the person is responsible for program planning and takes the leadership role for the entire program from the application to conduction. The main performer assists the leader in carrying out the program, and the participants implement intervention measures in CHCs. The assessor evaluates the investment and output of the program. A GP may or may not participate in one or more evidence-based programs for chronic diseases at their discretion. To facilitate the respondent's recall of the number of participating programs, we set 15 chronic disease categories (such as diabetes, hypertension, and chronic obstructive pneumonia, also, they can fill in the specific chronic diseases not in the categories) and asked them to fill in whether they participated in the programs and their role in the programs. Finally, we counted the total number of programs involved in all categories as the number of chronic disease programs participated in by GPs. The number of evidence-based programs for various diseases was analyzed first as the dependent variable to assess the overall effects of the impeding factors. Furthermore, we used the variable of whether respondents had held the leadership role to explore the key influencing factors for GP leaders.

### Statistical Analysis

SPSS 22.0 was used for statistical analyses. The demographics of the respondents were summarized using the mean and SD for continuous variables and frequency and percentages for categorical variables. Regarding the number of evidence-based programs attended by GPs, ordinal logistic regression was used to identify possible influencing factors. In terms of whether the GPs had held the leadership position, a binary logistic regression was utilized to identify various related levels of factors. Associations were measured by odds ratios (ORs) with 95% CIs. *p* < 0.05 was considered statistically significant.

## Results

### Characteristics of the Respondents

A total of 892 GPs, 50.9% of whom were women, responded to our survey. As shown in Table 2, the average age is 37.23 years. Most of them (77.7%) had a bachelor's degree. Most of the GPs in our study had mid-level (45.7%) and junior (46.6%) titles. Policy and economic factors were the most reported impediments, with an average value of 4.17, followed by external environmental factors, with an average value of 4.12. Personal and organizational factors had relatively lower average values of 3.99 and 3.82, respectively, which means that the barriers caused by these factors were lower than those of the policy and economic factors and external environmental factors on average. Moreover, as seen in Table 3, at the personal level, “not enough time” has the highest average score of 4.50, which means it is a moderate to the relatively large impediment. “Lack of decision-making authority” (mean = 4.23) scored second on the individual level. “Not enough staff assisted” had the highest score of 4.42 at the organizational level, followed by “not enough funding” (mean = 4.16) and “lack of access to evidence” (mean = 3.82). “Local residents' perception conflicts with evidence-based recommendations” (mean = 4.19) and “distrust of scientific data in the populations served” (mean = 4.14) received the highest scores at the external environmental level. “Not enough financial support from the government” (mean = 4.31) and “funding changes as political leadership changes” (mean = 4.26) received higher scores than “no existing policies to support evidence-based approaches” (mean = 4.13) and “policy climate conflicts with evidence-based recommendations” (mean = 4.07) at the policy and economic levels. Moreover, we found that 346 (38.8%) of the GPs had never undertaken or participated in any EBP program, while 148 (16.6%) had participated in four or more.

**Table 2:** Characteristics and perceived impeding factors of the respondents (*n* = 892).

**Characteristic**	**Mean ±SD**	***N* (%)**
**Demographics**		
**Gender**		
Female		454 (50.9)
Male		438 (49.1)
Age (years)	37.23 ± 7.37	
**Education**		
Associate's degree or below		89 (10.0)
Bachelor's degree		693 (77.7)
Master's degree or higher		110 (12.3)
**Employment title**		
Junior		416 (46.6)
Mid-level		408 (45.7)
Senior		68 (7.6)
Working years (years)	10.04 ± 8.16	
**Monthly income (RMB)**		
≤ 6,000		344 (38.6)
6,001–9,000		385 (43.2)
≥9,000		163 (18.3)
**Department**		
General medicine (Western medicine)		478 (53.6)
Prevention and healthcare		228 (25.6)
General practice (traditional Chinese medicine)		91 (10.2)
Other departments		95 (10.7)
**Area**		
Urban		484 (54.3)
Suburban		408 (45.7)
**Whether affiliated with a university**		
No		702 (78.7)
Yes		190 (21.3)
**Number of evidence-based programs attended**		
0		346 (38.8)
1		149 (16.7)
2		144 (16.1)
3		105 (11.8)
≥4		148 (16.6)

**Table 3:** Scores of various contextual factors (*n* = 892).

**Factor**	**Mean ±SD**	**Factors**	**Mean (95% CI)**
Personal factors	3.99 ± 0.96	Low value of evidence-based approaches	3.28 (3.19, 3.37)
		Lack of skills to find evidence	3.86 (3.78, 3.93)
		Lack of skills to develop evidence-based interventions	3.93 (3.85, 4.00)
		Lack of decision-making authority	4.23 (4.15, 4.31)
		Not enough time	4.50 (4.41, 4.58)
Organizational factors	3.82 ± 1.10	Poor understanding of evidence-based approaches	3.52 (3.44, 3.60)
		Culture/climate is not supportive of change	3.60 (3.51, 3.68)
		Leadership does not care about EBPs	3.52 (3.44, 3.61)
		Lack of internal policy to ensure interventions are evidence-based	3.71 (3.63, 3.80)
		Organization does not provide training in evidence-based approaches	3.59 (3.51, 3.68)
		Lack of access to evidence	3.82 (3.73, 3.91)
		Not enough funding	4.16 (4.07, 4.26)
		Not enough staff to assist	4.42 (4.32, 4.51)
External environmental factors	4.12 ± 1.00	Distrust of scientific data in the populations served	4.14 (4.06, 4.22)
		Local residents' perception conflicts with evidence-based recommendations	4.19 (4.11, 4.26)
		Insufficient relevant evidence for population served	4.11 (4.03, 4.18)
		Community members' needs conflict with evidence-based recommendations	4.11 (4.03, 4.18)
Policy and economic factors	4.17 ± 1.10	Insufficient financial support from the government	4.31 (4.23, 4.40)
		Funding changes as political leadership changes	4.26 (4.18, 4.34)
		Policy climate conflicts with evidence-based recommendations	4.07 (3.00, 4.15)
		No existing policies to support evidence-based approaches	4.13 (4.04, 4.21)

### Participation in Evidence-Based Chronic Disease Programs

Table 4 indicates that the evidence-based programs in which the GPs participated were predominantly related to hypertension, diabetes, and cardiovascular disease. Approximately 60% of the practitioners had participated in diabetes evidence-based preventative interventions. However, the proportion of GPs in the leadership role was relatively low, between 0.8% (programs involving asthma prevention and control) and 5.0% (diabetes). We also found that programs related to maternal and child health (1.2%), student health (1.4%), and arthritis (1.0%) had fewer GPs serving in the leadership role.

**Table 4:** The number of evidence-based programs attended and roles in specific chronic disease fields (*n* = 892).

**Field**	**Number of respondents in each position [*****n*** **(%)]**				
	**Leader**	**Main**	**Participant**	**Assessor**	**None**
		**performer**			
Diabetes	45 (5.0)	118 (13.2)	387 (43.4)	84 (9.4)	361 (40.5)
Hypertension	34 (3.8)	112 (12.6)	385 (43.2)	87 (9.8)	370 (41.5)
Tumor	26 (2.9)	60 (6.7)	292 (32.7)	60 (6.7)	512 (57.4)
Cardiovascular disease	22 (2.5)	81 (9.1)	337 (37.8)	70 (7.9)	446 (50.0)
Overweight and obesity	18 (2.0)	54 (6.1)	264 (29.6)	73 (8.2)	543 (60.9)
Diet/nutrition	18 (2.0)	63 (7.1)	233 (26.1)	44 (4.9)	577 (64.7)
Tobacco control	16 (1.7)	54 (6.1)	292 (32.7)	51 (5.7)	518 (58.1)
COPD	15 (1.7)	52 (5.8)	274 (30.7)	60 (6.7)	539 (60.4)
Eye protection	15 (1.7)	34 (3.8)	238 (26.7)	46 (5.2)	586 (65.7)
Student health	12 (1.4)	38 (4.3)	245 (27.5)	46 (5.2)	578 (64.8)
Osteoporosis	12 (1.4)	39 (4.4)	233 (26.1)	45 (5.0)	598 (67.0)
Maternal and child health	11 (1.2)	43 (4.8)	219 (24.6)	44 (4.9)	605 (67.8)
Arthritis	9 (1.0)	34 (3.8)	208 (23.3)	58 (6.5)	607 (68.1)
Asthma	7 (0.8)	40 (4.5)	220 (24.7)	48 (5.4)	603 (67.6)

### Influencing Factors of EBPs and the Leadership Role in Programs

Table 5 shows that those who perceived higher scores for personal factors (OR = 0.84, *p* = 0.05) and organizational factors (OR = 0.76, *p* < 0.01) attended fewer evidence-based programs. However, the results show that the higher the scores of policy and economic impediments were, the greater the number of evidence-based programs the GPs had attended (OR = 1.21, *p* = 0.03). In addition, compared with GPs in suburban areas, those from urban areas had attended more programs (OR = 1.45, *p* < 0.01). Additionally, compared with GPs practicing in Western medicine departments, GPs in prevention and healthcare departments (OR = 0.72, *p* = 0.03) and other departments (OR = 0.44, *p* < 0.01) had attended fewer evidence-based programs.

**Table 5:** Logistic regression of evidence-based practice and whether the GPs held the leadership position in the program (*n* = 892).

	**Number of evidence-based programs attended (*****n*** **=** **892)**			**Held the leadership role (*****n*** **=** **892)**		
**Variable**	**Sig**.	**OR**	**95% CI**	**Sig**.	**OR**	**95% CI**
**Contextual impeding factors**						
Personal factors	0.05	0.84	(0.70, 1.00)	0.13	0.77	(0.55, 1.08)
Organizational factors	<0.01	0.76	(0.64, 0.91)	0.81	0.96	(0.69, 1.34)
External environmental factors	0.38	1.09	(0.90, 1.30)	0.10	0.75	(0.53, 1.05)
Policy and economic factors	0.03	1.21	(1.02, 1.43)	0.01	1.47	(1.09, 1.98)
**Demographics**						
**Gender**						
Female		1.00			1.00	
Male	0.79	0.12	(0.81, 1.31)	0.02	1.73	(1.09, 2.74)
Age	0.11	1.02	(1.00, 1.04)	0.01	0.94	(0.90, 0.99)
**Education**						
Master's degree or higher		1.00			1.00	
Bachelor's degree	0.05	1.48	(1.00, 2.20)	0.74	1.14	(0.53, 2.43)
Associate's degree or below	0.11	1.60	(0.89, 2.87)	1.00	1.00	(0.33, 2.98)
**Employment title**						
Senior		1.00			1.00	
Mid-level	0.74	1.09	(0.66, 1.79)	0.29	0.59	(0.23, 1.55)
Junior	0.60	1.17	(0.66, 2.05)	0.46	0.66	(0.22, 1.97)
**Monthly income (RMB)**						
≤ 6,000		1.00			1.00	
6,001–9,000	0.27	0.86	(0.65, 1.13)	0.27	1.36	(0.79, 2.33)
≥9,000,	0.42	1.15	(0.81, 1.64)	<0.01	2.59	(1.36, 4.90)
**Department**						
General medicine (Western medicine)		1.00			1.00	
Prevention and healthcare	0.03	0.72	(0.53, 0.97)	<0.01	3.51	(2.13, 5.78)
General practice (traditional Chinese medicine)	0.08	0.69	(0.45, 1.05)	0.08	0.33	(0.10, 1.12)
Other departments	<0.01	0.44	(0.29, 0.68)	0.19	0.48	(0.16, 1.44)
**Area**						
Suburban		1.00			1.00	
Urban	<0.01	1.45	(1.12, 1.90)	0.61	1.14	(0.69, 1.87)
**Whether affiliated with a university**						
No		1.00			1.00	
Yes	0.22	1.21	(0.89, 1.66)	0.38	1.29	(0.74, 2.26)

Regarding whether the GPs had held leadership roles, the binary logistic regression indicated that GPs who had taken leadership roles perceived higher scores for policy and economic impeding factors (OR = 1.47, *p* = 0.01). As their age increased, the GPs were less likely to take leadership roles (OR = 0.94, *p* = 0.01). Additionally, those who were men (OR = 1.73, *p* = 0.02) and had a higher income (OR = 2.59, *p* < 0.01) were more likely to play a leadership role. Regarding the GPs' departments, those in prevention and healthcare departments (OR = 3.51, *p* < 0.01) were more likely to be responsible for evidence-based programs than those in general medicine (Western medicine).

## Discussion

In our study, we found that GPs were the leaders in and participated in more evidence-based programs for preventing hypertension, diabetes, and cardiovascular disease. Comparatively, other diseases, such as asthma, arthritis, maternal and child health, and student health, have received less attention. The reason for this result may be related to disease prioritization, specifically in China. Although younger and older populations are both target populations, more attention is given to the more prevalent diseases of hypertension, diabetes, and cardiovascular disease (19, 20). Nevertheless, it cannot be denied that GPs should pay more attention to evidence-based programs for younger populations, as studies have also revealed that obesity and mental health are prevalent and increasing disease categories (21, 22). In our study, we also found that 38.8% of the GPs had not participated in any EBP program for chronic disease prevention. In the past two decades, organizations, such as the clinical epidemiology committee of the Chinese Medical Association and the Chinese Cochrane Center, have developed programs for advanced practitioners, such as GPs' utilization of evidence-based principles (23). However, EBPs have not been widely disseminated (24).

Evidence-based practice is a relatively novel concept for most Chinese GPs, and they may not have studied them systematically (25). Additionally, compared with the United States and Australia, practitioners from China were found to know less about EBPs (17, 26). Although capacity-building programs for public health practitioners are widely conducted in developed countries, homogeneous programs are rather scarce in developing countries such as China (10, 17). In such a context, training programs are needed to improve GPs' capability of conducting evidence-based programs. In this investigation, training courses, lectures, and seminars were mentioned many times in the open-ended questions to raise individual evidence-based awareness of and build capabilities for EBPs.

Regarding the impeding factors, we found that personal, organizational, political, and economic factors exerted a significant effect on the number of evidence-based programs that GPs had attended. Regarding personal factors, we found that GPs perceived a lack of decision-making authority and not having enough time as highly impeding subfactors. The reasons may be the following: (1) China's top-down tiered healthcare delivery system determines that GPs in primary healthcare systems do not have much decision-making authority in either clinical or population-based health interventions. When dealing with complicated and severe medical disorders, they need to refer patients to superior hospitals. When carrying out interventions in communities, GPs need to obtain permission from leaders and cooperation from community neighborhood committees before obtaining funding from the government (3, 27). (2) After the New Health Reform in 2009, the government paid increasing attention to CHCs, and GPs gradually became the main providers of primary care in cities (28). Outpatient and scientific research occupied most GPs' working hours, and many of them chronically lacked time (29).

In our study, we found that organizational factors are also impediments to EBPs, and a lack of internal policy to ensure that interventions in the organization are evidence-based should be considered. GPs will not use evidence-based approaches or participate in any evidence-based programs if they are not encouraged or rewarded for doing so. A system should be developed to maintain GPs' motivation EBPs. “Not enough staff assisted” is another factor worth noting and coexists with “lack of time” at the personal level. The primary health system faces a critical shortage of qualified GPs due to various issues, such as insufficient training and less pay than specialists (3, 30). Widespread low job satisfaction and high occupational burnout among GPs have become challenges for the strengthening of China's primary healthcare system and exacerbated the shortage of CHC staffing (3, 31). Access to evidence is also worthy of attention. In China, most medical databases, such as the two largest Chinese medical databases, CNKI, and Wanfang Database, are not freely accessible to the public (25). GPs from large CHCs have better database permissions and can participate in more academic conferences (23). However, small CHCs in suburban areas do not have the funding to purchase expensive medical database access. To overcome this dilemma, systematic reviews and guidelines should be compiled and made available to GPs in local CHCs. Existing studies have indicated that improving certain organizational processes can facilitate EBPs and promote agency performance (12, 32). Administrative EBPs (A-EBPs), a set of core competencies for public health administrators, are agency-level structures and activities that are positively associated with performance measures (12, 32, 33). In developed countries, some capacity-building courses for chronic disease practitioners in the early stage tend to focus on the discovery and appraisal of evidence but place less emphasis on A-EBPs (34). Organizational factors may be more difficult to intervene in, but they sometimes cause greater impediments than individual capabilities (13, 35). Working atmosphere construction, workforce development, and access to evidence all follow leaders' understanding and appreciation of EBPs. Recently, a leadership competency framework was developed to support the curriculum aimed at leadership (36, 37). Predictably, in addition to training courses for chronic disease practitioners, leadership expertise building for both technique and management is critical for the promotion of EBPs.

We found that the policy and economic factors significantly influenced both the number of evidence-based programs in which the GPs had participated and whether they had taken the leadership role. Additionally, these subfactors had the highest scores. Among them, “not enough financial support from government” had the highest score. Financial constraints are always impediments to EBP implementation (11, 25, 33, 34). In the context of a high incidence of chronic disease, a significant amount of funding is required to conduct evidence-based preventative interventions. Emphasis on research and the neglect of translation from research to practice have intensified the funding shortages in the practice of chronic disease prevention (11). The publication of studies is not the end of the disease prevention process (15), and it is necessary to communicate to policy makers what EBPs for chronic disease are, why they are important, and why funds for implementing preventative interventions are needed to complete the whole EBP process (11).

However, it was interesting that GPs who had participated in fewer evidence-based programs were more likely to report personal and organizational factors, while GPs who had participated in more evidence-based programs and played a leadership role in the programs were more likely to consider policy and economic factors to be greater impediments. If personal and organizational factors, such as “lack of capacity to develop evidence-based interventions” and “lack of internal policy to ensure interventions are evidence-based”, were overcome, policy and economic impediments tend to become bottlenecks in GPs' implementation of EBPs.

Compared with participating in evidence-based programs, whether the GPs had held a leadership role was more likely to be influenced by demographic characteristics. Male GPs, those with a higher monthly income, those from urban areas, and younger GPs, were more likely to have been in the leadership role in evidence-based programs. However, these factors have no significant influence on the number of evidence-based programs they had participated. Both the number of evidence-based programs and whether the GPs had held the leadership role differed between different departments. Compared with GPs from general medicine (Western medicine) departments, GPs from prevention and healthcare departments had participated in fewer EBP programs but were more likely to have taken the leadership role. Although GPs from prevention and healthcare departments dominated the evidence-based programs, the influence of the programs was limited, and the programs attracted fewer of these GPs than those from general medicine (Western medicine) departments. More resources need to be allocated to the prevention process to promote EBPs.

The limitations of this study should be noted. First, Shanghai is located in China's economically developed region, and GPs' EBPs may therefore be better there than in the central and western regions of China. Second, the content of the questionnaire was mainly subjective, which may cause bias. Finally, all data were self-reported, and it was difficult to verify the accuracy.

## Conclusion

Evidence-based programs for chronic diseases should be extended to address the types of diseases encountered by GPs. Capacity-building courses are needed to help GPs find and translate evidence into practice in China. More resources should be allocated to GPs, especially those who are men, have a lower income, and live in suburban areas. GPs from prevention and healthcare departments should be given more opportunities to take leadership roles compared with those from Western medicine departments. Moreover, efforts should be made to overcome the difficulties of a lack of staff and insufficient time of GPs. Internal policy development and leadership expertise building should be accelerated to create an appropriate environment for EBPs. Policy and funding support is needed to facilitate the generation of more high-quality evidence and implementation of preventative interventions at the population level in the future.

## Data Availability Statement

The original contributions presented in the study are included in the article/supplementary material, further inquiries can be directed to the corresponding authors.

## Ethics Statement

The studies involving human participants were reviewed and approved by Tongji University (Ref: LL-2016-ZRKX-017). The patients/participants provided their written informed consent to participate in this study.

## Author Contributions

All authors made a significant contribution to the work-reported, whether that is in the conception, study design, execution, acquisition of data, analysis, and interpretation, or in all these areas, took part in drafting, revising, or critically reviewing the article, gave final approval of the version to be published, have agreed on the journal to which the article has been submitted, and agree to be accountable for all aspects of the work.

## Funding

The design of this study involving some previous investigation was supported by the Shanghai Excellent Young Talents Project in Health System (2018YQ52). Data extraction was funded by the Natural Science Foundation of China (71774116 and 71603182) and the Shanghai Public Health System Construction Three-Year Action Plan (GWV-10.1-XK15). The analysis and interpretation of the data guided by the statisticians were funded by grants from the National Key R&D Program of China (2018YFC2000700). The writing and revision, including the language improvement, were supported by Shanghai Pujiang Program (2019PJC072).

## Conflict of Interest

The authors declare that the research was conducted in the absence of any commercial or financial relationships that could be construed as a potential conflict of interest. The reviewer YS declared a shared affiliation with some of the authors XGa, HJ, NC, YY, and JS to the handling editor at time of review.

## Publisher's Note

All claims expressed in this article are solely those of the authors and do not necessarily represent those of their affiliated organizations, or those of the publisher, the editors and the reviewers. Any product that may be evaluated in this article, or claim that may be made by its manufacturer, is not guaranteed or endorsed by the publisher.

## Abbreviations

GPs: General practitioners
CHCs: Community healthcare centers
COPD: Chronic obstructive pulmonary disease
EBPs: Evidence-based practices
CHAMPS: Community healthcare for asthma management and prevention of symptoms.
